# K-line guided approach selection and outcomes assessment for Cervical Ossification of Posterior Longitudinal Ligament (OPLL): A case series with literature review from Pakistan

**DOI:** 10.12669/pjms.40.12(PINS).11094

**Published:** 2024-12

**Authors:** Haseeb Mehmood Qadri, Manal Khan, Nasruddin Ansari, Ahtesham Khizar, Syed Faizan Ahmad Bukhari, Asif Bashir

**Affiliations:** 1 Haseeb Mehmood Qadri, MBBS Postgraduate Resident Neurosurgery, Department of Neurosurgery Unit-I, Punjab Institute of Neurosciences, Lahore, Pakistan; 2 Manal Khan, MBBS Postgraduate Resident Neurosurgery, Department of Neurosurgery Unit-I, Punjab Institute of Neurosciences, Lahore, Pakistan; 3 Nasruddin Ansari, MBBS Postgraduate Resident Neurosurgery, Department of Neurosurgery Unit-III, Punjab Institute of Neurosciences, Lahore, Pakistan; 4 Ahtesham Khizar, MBBS, FCPS Senior Registrar Neurosurgery, Department of Neurosurgery Unit-I, Punjab Institute of Neurosciences, Lahore, Pakistan; 5 Syed Ahmad Faizan Bukhari, MBBS, FCPS Assistant Professor Neurosurgery, Department of Neurosurgery Unit-I, Punjab Institute of Neurosciences, Lahore, Pakistan; 6 Asif Bashir, MD, FAANS, FACS Professor of Neurosurgery, Department of Neurosurgery Unit-I, Punjab Institute of Neurosciences, Lahore, Pakistan

**Keywords:** Ossification of Posterior Longitudinal Ligament, Posterior Longitudinal Ligament, Myelopathy, Laminectomy, Laminoplasty, Developing country

## Abstract

**Objectives:**

To analyze the efficacy of K-line in surgical planning of approach selection for ossification of posterior longitudinal ligament (OPLL) and outcomes assessment by Nurick grading and Modified Japanese Orthopaedic Association (mJOA) scores.

**Methods:**

This is a retrospective case series study conducted at the Departments of Neurosurgery, Punjab Institute of Neurosciences, Lahore in the months of January and February 2024. Patients with complete records were considered. Google Form was used for data. Nurick grading and Modified Japanese Orthopaedic Association (mJOA) scores were calculated for each patient pre- and post-operatively. K-line assessment was done on computerized tomography (CT). Data analysis was performed using Microsoft excel in terms of frequency and percentages.

**Results:**

This study included ten patients with the mean age at presentation was 48.20 ± 9.37 years. Preoperative Nurick grading in our patients was Grade-V (five patients), Grade-IV (two patients) and Grade-III (three patients) with mean of 4.2 ± 0.91 SD whereas six months follow-up Nurick grading was Grade-V (three patients), Grade-IV (three patients) and Grade-III (four patients) with the mean of 3.90 ± 0.87 SD, which indicates neurological improvement. Types of OPLL present in our patients were segmental (4, 40%), continuous (3, 30%), mixed (2, 20%) and localized/others (1, 10%). There were 2 (20%) K-line (+) and 8 (80%) K-line (-) cases. Anterior approach was used in 2 (20%) cases whereas posterior approach was used for the rest of 8 (80%) cases. Mean preoperative mJOA was 10.30 ± 2.45 and mean postoperative mJOA was12.40 ± 2.01 at 6-month follow up, which indicates improvement in our cases.

**Conclusion:**

K-line is a useful radiological indicator in selecting anterior versus posterior approach for patients with cervical OPLL in terms of Nurick grading and mJOA scores.

**List of Abbreviations:**
OPLL: Ossified Posterior Longitudinal Ligament,
DISH: Diffuse Idiopathic Skeletal Hyperostosis,
CT: Computed Tomography,
mJOA: Modified Japanese Orthopaedic Association,
BMP: Bone Morphogenetic Protein,
TGF-b: Transforming Growth Factor Beta,
ACCF: Anterior Cervical Corpectomy and Fusion.

## INTRODUCTION

Ossification of Posterior Longitudinal Ligament (OPLL) is a multifactorial disease involving abnormal calcification of the posterior longitudinal spinal ligament causing spinal cord compression leading to myelopathy and compromised neurology. OPLL frequently affects cervical spine (70%) involving C5 segment.^1^ It occurs after 40 years of age with males affected twice as much as females. Japanese people have the highest prevalence of OPLL about 1.9-4.3% followed by 0.8-3% of South East Asians and then 0.1-1.7% of North Americans and Europeans. Asian Americans have the highest prevalence of 4.8% and Caucasians Americans 1.3% the lowest. The etiology of OPLL is unknown. It is affected by genetic factors, environmental factors, metabolic factors affecting calcium homeostasis viz hypoparathyroidism/acromegaly/osteomalacia and others being elderly age, uncontrolled blood sugar, increased body weight, and sedentary lifestyle.^2^ OPLL is attributable to multiplication and transformation of fibroblast like chondrocyte and osteoblasts along with formation of new blood vessels within the ligament caused by Bone Morphogenic Protein (BMP) overexpression in the calcified spinal ligaments. This leads to formation of cartilaginous tissue with hyaline degeneration and calcification in the center. Progression of OPLL is silent and slow but may present acutely following trivial trauma with cervical myelopathy symptoms.^3^

Progressively increasing neurological deficit in OPLL needs surgical attention. Multiple radiological criteria have been developed for classifying OPLL for surgical planning. It includes myelopathic changes in the spinal cord as a result of dynamic factors such as listhesis or hypermobility at segmental or mixed type of OPLL or in fractured ossified ligament, number of spinal segments involved along with involvement of atlas cervical vertebra, measurement of cervical sagittal balance and K-line, correlating between OPLL and C2-C7 line.^4^ The K-line is a virtual line connecting the antero-posterior diameter of the spinal canal from C2-C7 on a plain lateral X-ray film or computed tomography (CT). OPLL going beyond this line is K-line negative otherwise, it is K-line positive. It is an important prognostic tool to assess adequate decompression by laminoplasty for OPLL. K-line negative patients will not be benefitted by just posterior decompression.^3^

There is scarcity of literature on OPLL from Pakistan. We have very little documentation of cases on OPLL. These studies have not considered K-line as an important factor for deciding surgery other than clinical factors which are also paramount things to decide whether to operate or not considering the crippling nature of the disease. Therefore, we designed this case series to analyze the efficacy of K-line in surgical planning of OPLL patients in our setup.

## METHODS

This is a retrospective case series conducted at the Departments of Neurosurgery, Punjab Institute of Neurosciences, Lahore in the months of January and February 2024.

### Ethical Approval:

Institutional review board provided approval for this study with the reference No. 1784/IRB/PINS/Approval/2024.

Patients operated for cervical OPLL between January 01, 2022 and December 31, 2023 were studied using non-probability, consecutive sampling. All operated patients with complete records and the diagnosis of cervical OPLL were considered, regardless of age and gender. Patients with OPLL in regions other than the cervical spine or those with incomplete records were excluded from this study. Google Form (Google IncMountainview, CA) was used as a data collection instrument, covering various variables such as demographic details, clinical presentation, findings of central nervous system examination and radiological investigations, type of surgical procedures for OPLL and post-operative complications. Nurick grading and Modified Japanese Orthopaedic Association (mJOA) scores were calculated for each patient pre- and post-operatively. K-line assessment was done on CT scans. Data analysis was performed using Microsoft excel in terms of frequency and percentages. Additionally, PubMed, Google scholar and PakMediNet were searched for existing literature from Pakistan. The search terms used were “OPLL” AND “Pakistan” and “cervical OPLL” AND “Pakistan”. Five articles were retrieved from these databases and are described in review of the literature.

## RESULTS

For the included ten patients, with eight males and two females, the mean age at presentation was 48.20 ± 9.37 years. Risk factors present in patients with cervical OPLL were Diffuse Idiopathic Skeletal Hyperostosis (DISH), trauma and obesity in two (20%), two (20%) and one (10%) patient, respectively, (Table-I). The higher the Nurick grading, the worse are the symptoms, and vice versa. Preoperative Nurick grading in our patients was Grade-V (five patients), Grade-IV (two patients) and Grade-III (three patients) with mean of 4.2 ± 0.91 SD whereas at 6-month follow-up Nurick grading was Grade-V (three patients), Grade-IV (three patients) and Grade-III (four patients) with the mean of 3.90 ± 0.87 SD, which indicates neurological improvement, (Table-II).

**Table-I:** Risk factors present in patients with cervical OPLL.

Sr. #	Risk Factors	Number of Patients, n	Percentage Occurrence, n/N
1.	Diffuse Idiopathic Skeletal Hyperostosis	2	20%
2.	Trauma	2	20%
3.	Obesity	1	10%

**Table-II:** Preoperative and six months follow-up Nurick grading of patients with cervical OPLL.

Sr. #	Nurick Grading	Number of Patients on presentation	Number of patients on six months follow up
1.	Grade-V	5	3
2.	Grade-IV	2	3
3.	Grade-III	3	4
Mean ± SD		4.2 ± 0.91	3.90 ± 0.87

**Fig.1:**
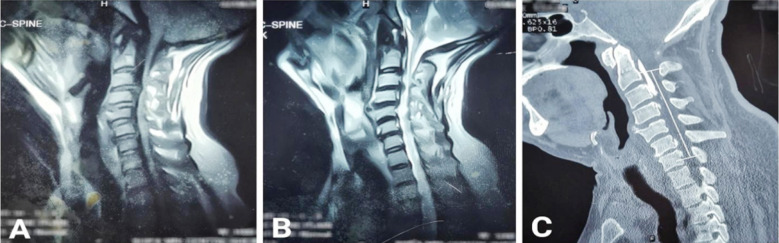
A: MRI cervical spine sagittal T1WI showing hypointense lesion in PLL region at C1, C2 & C3, B: MRI cervical spine sagittal T2WI showing hypointense lesion in PLL region at C1, C2 & C3, C: CT cervical spine sagittal view showing localized type of OPLL at C1, C2 & C3.

**Fig.2:**
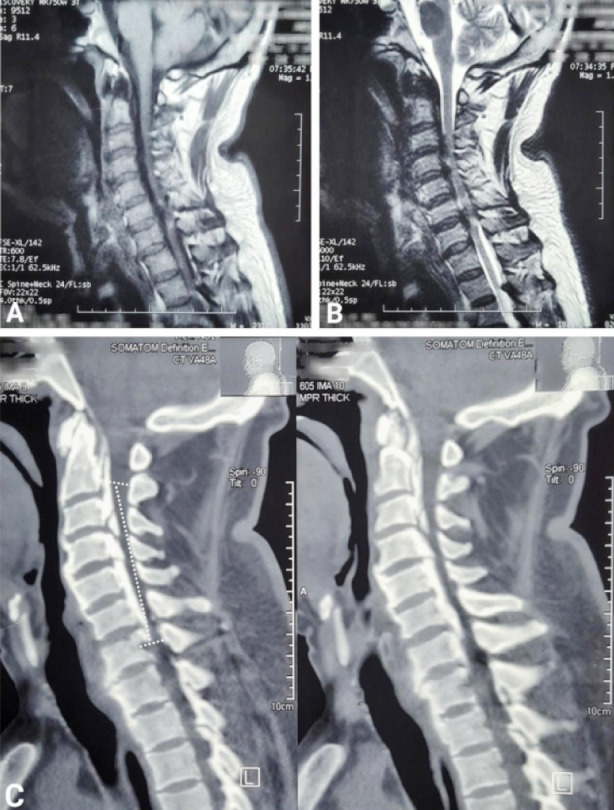
A: MRI cervical spine sagittal T1WI showing hypointense lesion in PLL region from C2 to C7, B: MRI cervical spine sagittal T2WI showing similar lesion from C2 to C7, C: CT cervical spine sagittal view showing mixed type of OPLL from C2 to C7.

**Fig.3:**
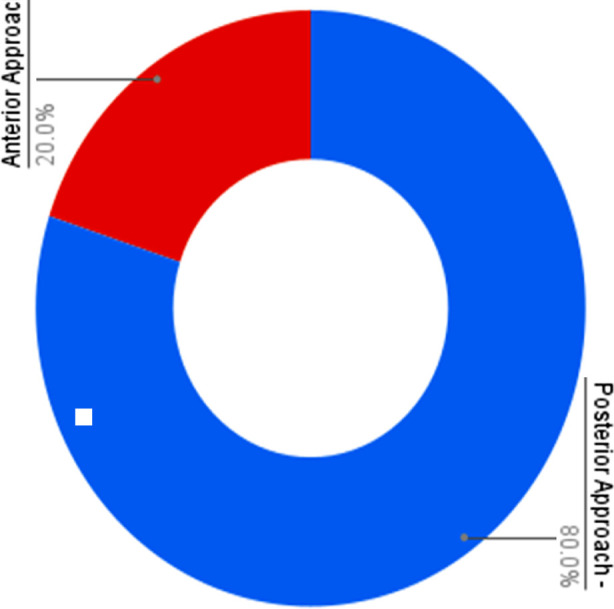
Type of surgical approach in patients with cervical OPLL.

Mean duration of presenting complaints was 8.50 ± 5.81 months. Involved segments with cervical OPLL were C3-C6 (3, 30%), C3-C5 (2, 20%), C2-C3 (1, 10%), C2-C5 (1, 10%), C2-T1 (1, 10%), C3-C4 (1, 10%) and C5-C7 (1, 10%), (Table-III). Types of OPLL present in our patients were segmental (4, 40%), continuous (3, 30%), mixed (2, 20%) and localized/others (1, 10%), (Table-IV). Fig.1 shows a localized type of OPLL in one of our cases while Fig.2 shows a mixed type of OPLL. There were two (20%) K-line (+) and 8 (80%) K-line (-) cases. (Table-V) Anterior approach was used in two (20%) cases whereas posterior approach was used for the rest of 8 (80%) cases. (Fig.3) Mean preoperative mJOA was 10.30 ± 2.45 and mean postoperative mJOA was12.40 ± 2.01 at 6-month follow up, which indicates improvement in our cases, (Table-VI).

**Table-III:** Levels of involved segments in patients with cervical OPLL.

Sr. #	Levels of involved segments	Number of Patients, n	Percentage Occurrence, n/N
1.	C3-C6	3	30%
2.	C3-C5	2	20%
3.	C2-C3	1	10%
4.	C2-C5	1	10%
5.	C2-T1	1	10%
6.	C3-C4	1	10%
7.	C5-C7	1	10%

**Table-IV:** Types of OPLL present in patients with cervical OPLL.

Sr. #	Type of OPLL	Number of Patients, n	Percentage Occurrence, n/N
1.	Segmental	4	40%
2.	Continuous	3	30%
3.	Mixed	2	20%
4.	Localized/Others	1	10%

**Table-V:** Assessment of K-line in patients with cervical OPLL.

Sr. #	K-line status	Number of Patients, n	Percentage Occurrence, n/N
1.	K line (+)	2	20%
2.	K line (-)	8	80%

**Table-VI:** Pre- and postoperative modified Japanese Orthopedic Association scores (mJOA) in patients with cervical OPLL.

Patients	Preoperative mJOA					Postoperative mJOA				

	Motor U/E	Motor L/E	Sensory U/E	Sphincters	Total	Motor U/E	Motor L/E	Sensory U/E	Sphincters	Total
Patient 1	2	2	1	2	7	3	3	2	2	10
Patient 2	4	5	2	3	14	4	5	3	3	15
Patient 3	4	6	1	3	14	4	6	2	3	15
Patient 4	2	2	3	2	9	3	4	3	2	12
Patient 5	2	5	1	1	9	3	5	2	1	11
Patient 6	2	2	3	0	7	3	3	3	1	10
Patient 7	3	2	3	3	11	4	3	3	3	11
Patient 8	3	2	3	3	11	3	3	3	3	12
Patient 9	3	2	3	2	10	4	4	3	2	13
Patient 10	2	4	2	3	11	4	4	4	3	15
Mean ± SD	10.30 ± 2.45					12.40 ± 2.01				

**Table-VII:** Review of the existing scientific literature from Pakistan using PubMed, Google Scholar and PakMediNet.

Characteristics of Study	Chaudhry et al.^6^	Younus et al.^8^	Khalid et al.^9^	Khulsai et al.^7^
Year of Publication	2012	2015	2017	2018
Cohort Size	3	20	10	18
Average Age (years)	-	57.45	43.44	52
Gender Distribution	2 M, 1 F	16 M, 4 F	-	15 M, 3 F
Average Duration of Presenting Complaints (months)	-	19.70	-	14.5
Involved Segments of Cervical Spine	-	C3: 1 C3 - C5: 2 C3, C4: 4 C4: 1 C4, C5: 12	-	C3 - C5: 6 C4 - C6: 9 C5 - C6: 3

## DISCUSSION

Long thought of as the disease of East Asians, cases of ossified posterior longitudinal ligament (OPLL) are being documented increasingly from Pakistan and India.^5^-^9^ The myth “Japanese disease” does not hold true anymore. Singh et al. document a prevalence of 5.12% of cervical OPLL in their 11 years study on polytrauma patients from India, while Head et al. discuss a 12 years prevalence of 2.04% in Korea and a variable range of 1.9%-4.3% among various Japanese studies. The region of SouthEast Asia is an understudied area where OPLL in various segments of the spine has not been well-studied.^3^,^5^

For our included patients, the mean age of presentation was 48.20 ± 9.37 years. However, another study from our country by Younus et al. documents an average age of 57.45 years for the patients presenting with cervical OPLL.^8^ An Indian study in 2019 involving the patients of cervical OPLL also documents a mean age of presentation of 58.60 ± 7.8 years which is consistent with the findings of our study.^10^ The patients who presented at our center were more aware of their symptoms and contacted the hospital at an early onset of disease.

There were only two female patients in our case series, a finding similar to the study by Srivastva et al. where only 12.50% patients were female.^11^ Yet another Korean study by Moon et al. has 26.31% female patients emphasizing the fact that cervical OPLL affects males much more than female human beings.^12^

Transforming growth factor beta (TGF-b) and BMP play a pivotal role in differentiating mesenchymal stem cells into osteoblast, chondrocytes and adipocytes. The existing scientific literature proves their presence in ossified ligaments of the spine. Genome-wide association study of OPLL has brought attention to the six susceptibility loci, implying a familial pattern of OPLL etiogenesis.^2^,^3^ However, the patients we have studied in our case series are sporadic cases and have no history of OPLL or disorders of metabolism in their previous two generations.

Early onset of cervical OPLL is associated with increased severity at presentation.^2^ This is backed by the findings of our study where patients have presented in their fifth decade of life with 50% of them having Nurick Grade-V at presentation. Another Pakistani study documented in the province of Sindh shows 11 patients with Nurick Grade-IV and V at presentation in their early sixth decade of life.^7^

A higher level of evidence of American doctors has discussed the two schools of thoughts regarding the most frequent types of OPLL; one implying segmental type as the most frequently seen and the other signifying equal occurrence of segmental, continuous and mixed types.^4^ Dhillon et al. found 48.18% cases of segmental type in their cohort of 54 patients, while another study from the same country found mixed type to be the most common variety in 52.50% patients.^10^,^11^ We found segmental and continuous types in 40% and 30% cases respectively.

Multiple classification systems have been proposed to categorize cervical OPLL. Systems involving cross-sectional shape of spinal cord, dural ossification and rule of nine are comparatively cumbersome than K-line. The K-line is a one simple concept incorporating the kyphotic components of the cervical spine and the thickness of OPLL. K-line connects the midpoints of spinal canal cervical vertebrae, C2-C7. The maximum thickness of OPLL determines the status of K-line. Cases with maximum thickness beyond K-line are considered K-line positive (+).^3^,^4^ The existing English scientific literature suggests anterior approach, likely laminoplasty for K-line (+) cases of cervical OPLL, while reserving posterior approach, likely laminectomy and fusion K-line (-) patients.^4^ In agreement with the literature, we operated two K-line (+) cases using anterior approach and the remaining cases of K-line (-) were dealt with by posterior decompression. Nagoshi et al. conducted a multicentric, prospective study to compare the surgical outcomes of anterior and posterior fusion surgeries for K-line negative (−) cervical OPLL. Both approaches were equivocal in terms of functional and neurological outcomes.^13^ However, the study by Moon et al. shows anterior cervical corpectomy and fusion (ACCF) to be superior to posterior laminoplasty for cervical OPLL in the long-run in terms of neurological outcomes.^12^ There was significant improvement in mJOA from mean preoperative mJOA of 12.10 ± 0.72 to mean postoperative mJOA of 13.30 ± 0.91 in the sixth month. This is consistent with the similar findings of improved mJOA in an Indian study utilizing open door cervical laminoplasty for cervical OPLL.^10^

It is important to note that radiological parameters are not to be solely used to determine the surgical approach. Number of levels involved, preoperative neurology, accompanying comorbidities, surgeon’s preference and the risk of complications govern the final decision of surgical approach.^4^,^13^

### Limitations:

The retrospective nature, small sample size of the case series, with its unicentric nature limit the generalizability of the findings of this research study. K-line assessment was performed on CT scans, and not lateral radiographs. A naturally short follow-up period by patients of six months is another limiting factor.

## CONCLUSIONS

As per our study in terms of Nurick grading and mJOA scores, K-line appears to be a useful radiological predictor of whether to choose an anterior or posterior approach for patients with cervical OPLL. Moreover, surgical skills and patient characteristics should also be evaluated to enhance the K-line assessment in the selection of approach.

### Clinical recommendations:

K-line should be used as a standard radiological parameter to decide the optimal operative approach for cases of cervical OPLL. Surgical expertise and patient factors should be considered and aid the K-line assessment in choice of approach. Long-term follow up for operated cases of cervical OPLL is warranted to deduce solid conclusions regarding the comparative outcomes of opted techniques.

### Authors’ Contribution:

**HMQ:** Conception and design of study, data acquisition, manuscript writing and literature review. **MK and NA:** Data acquisition, manuscript writing and literature review. **AK:** Data analysis, Interpretation of data, and Manuscript editing. **SFAB and AB:** Supervision and critical review. All the authors have read and approved the final manuscript and are responsible and accountable for the accuracy and integrity of the work.
